# Volatile Organic Compounds Profile in White Sturgeon *(Acipenser transmontanus)* Caviar at Different Stages of Ripening by Multiple Headspace Solid Phase Microextraction

**DOI:** 10.3390/molecules25051074

**Published:** 2020-02-27

**Authors:** Annalaura Lopez, Mauro Vasconi, Federica Bellagamba, Tiziana Mentasti, Mario Pazzaglia, Vittorio Maria Moretti

**Affiliations:** 1 Department of Veterinary Medicine – Università degli Studi di Milano, Via dell’Università 6, 26900 Lodi, Italy; mauro.vasconi@unimi.it (M.V.); federica.bellagamba@unimi.it (F.B.); tiziana.mentasti@unimi.it (T.M.); vittorio.moretti@unimi.it (V.M.M.); 2 Agroittica Lombarda S.p.A. - Via J.F. Kennedy, 25012 Calvisano (BS), Italy; mario.pazzaglia@agroittica.it

**Keywords:** caviar, multiple headspace extractions, volatile organic compounds, flavour, SPME

## Abstract

Caviar is considered a delicacy by luxury product consumers, but few data are available about its flavour chemistry to date. In this study, a multiple headspace-solid phase microextraction (MHS-SPME) followed by gas chromatography and mass spectrometry (GC-MS) approach was developed and employed to identify and quantitatively estimate key volatile organic compounds (VOCs) representative in white sturgeon (*A. transmontanus*) caviar at five different stages of ripening: raw eggs (t0), after 60 days (t1), 120 days (t2), 180 days (t3), and 240 days (t4) of ripening. The method showed the ability to detect and estimate the quantity of 25 flavour compounds, without any severe alteration of the matrix before the analysis and in a short time. The VOCs detected as representative in caviar samples were primarily aldehydes and alcohols, already well known as responsible of fresh fish and seafood flavours, and mainly deriving from lipid peroxidation processes and microbial activity against lipids and amino acids. We found a significant (*p* < 0.01) increase in the amount of total aldehydes within t0 (29.64 ng/g) and t4 (121.96 ng/g); moreover, an interesting, great arise of 3-hydroxy-2-butanone at the final stage of storage (48.17 ng/g) was recorded. Alcohols were not detected in raw eggs (t0) and then a decrease from t1 (17.77 ng/g) to t4 (10.18 ng/g) was recorded in their amount, with no statistical significance.

## 1. Introduction

Caviar is defined as the product made from fish eggs of the *Acipenseridae* family by treating with food grade salt [1]. During the last 15 years, the presence on the market of caviar from aquaculture origin has increased and estimated to amount to 365 tons in 2017 [2], while the product coming from fisheries gradually disappeared on the legal market due to the global limit of capture of wild sturgeons. Sturgeons species are listed in Annex II and I of the Convention on International Trade in Endangered Species (CITES) [3] and, starting from 2006, CITES has no longer issued any quota for the marketing of caviar from wild stocks, thus catches for caviar production are completely forbidden nowadays. European sturgeon farmers in 2017 produced about 140 tons of caviar, with Italy as production leader with 43 tons, followed by France, Poland and Germany [2]. In such a scenario, the quality assurance and a solid characterisation of caviar as a precious product appear as fundamental issues for the safeguard of the Italian and European markets. Many analytical techniques have been developed during the years to assess caviar authenticity and quality factors. For instance, DNA testing, based on genetic interspecific divergences and variations, is a well-known technique used to verify the species source of the product to date [4,5,6], even if the presence of hybrid sturgeons could make it difficult to correctly attribute a caviar to a single species. Moreover, it has been demonstrated that the chemical composition of caviar can lead to discriminate, above all, between eggs obtained from farmed vs. wild sturgeons [7,8,9] or eggs coming from different species [10].

Caviar producers are very careful about the sensory evaluation that is generally carried out in-factory by people purposely trained and in accordance with the Guidelines for the Sensory Evaluation of Fish and Shellfish in Laboratories [11]. The Codex Alimentarius standard [1] stands that caviar samples affected by odour and/or flavour indicative of decomposition, oxidation, taste of feed (supplied to farmed sturgeon) or contamination by xenobiotic substances must be considered defective and cannot be addressed to human consumption. In such a context, the interest in developing analytical techniques to determine the characteristic volatile organic compounds (VOCs) profile of this “luxury delicacy” becomes consistent. It is known that in fresh fish and seafood very fresh flavours and aromas are characterised by mild, green and planty notes. The chemical basis of the fresh fish flavour is centred on the polyunsaturated fatty acids very representative in fish lipids. The major flavour impact compounds are several 6-, 8- and 9-carbon aldehydes, ketones and alcohols, which are derived from the fatty acids via specific lipoxygenase activity [12]. However, little information is available in the literature regarding caviar VOCs. Most data are obtained by extraction methods, such as simultaneous distillation–extraction (SDE), which is responsible for the formation of many artefacts, mainly due to the oxidation and thermal degradation of components during the extraction [9]. Such technique could be useful when the investigated food matrix consists of products that usually undergo cooking/heating processes, before human consumption. This is not the case of caviar, which is consumed raw, without any previous industrial process, just after the addition of low concentrations of food grade salt and at the end of an optimal ripening time (generally, five or six months) under refrigeration (at −2 °C).

Solid phase microextraction (SPME) is an analytical technique that allows a solvent-free extraction of analytes of interest developed in 1990 by Pawliszyn and Arthur [13]. SPME works by a partitioning process between the solid and the gaseous phase in the extraction chamber and then a partitioning process between the gaseous phase and a sorbent material, represented by a fused silica fibre coated with a thin layer of a selective coating. The fibre can be exposed to the sample matrix, in order to extract organic compounds of interest directly from the sample (direct immersion SPME) or from the sample headspace (HS). The extracted compounds are then desorbed and separated by HPLC or GC, often coupled with mass spectrometry (LC-MS and GC-MS) [14] and the signal intensity provided by SPME and GC-MS is proportional to the free concentration of target compounds, defining the fraction of the analyte that is bioavailable [15]. During last years, HS-SPME has been applied in many food analysis studies to detect the components responsible for the odour and aroma in a number of different food matrices [16,17,18,19]. However, HS-SPME is a non-exhaustive extraction method, since a determinate amount of analyte is removed by the sample matrix until its concentration reaches the equilibrium between the solid and gaseous phases involved in the process. Several approaches have been developed to overcome this issue and to reach a reliable quantification of extracted compounds. One of these is the so-called multiple headspace (MHS) extraction method, a stepped procedure whose theory was introduced by Kolb in 1982 [20]. Briefly, an almost exhaustive extraction of analytes is performed exposing the fibre to the sample HS in several consecutive extractions. After this step, a logarithmic linear regression is performed, plotting the number of performed extraction versus the natural logarithm of the respective total ion current (TIC) area for each compound. The slope of the linear regression line obtained represents the natural logarithm of β, where β is an analyte-dependent constant that indicates the extent of the decay across successive extractions, as follows:

(1) $$lnAi=ln\beta \;\left(i-1\right)+lnA1$$

with i the number of extraction steps, β the exponential decay of the chromatographic peak area, and A1 the area detected after the first extraction.

The β factors obtained in this step allow the estimation of the total area (A_tot_) for each analyte or, in other words, the area of the TIC for the analyte if the SPME would not be an equilibrium but an exhaustive extraction technique. In fact, dividing the area obtained after the first extraction by 1-β, it is possible to estimate the total cumulative response area for each compound through a geometric regression function, as shown in Equation (2):

(2) $$Atot=\frac{A1}{1-\beta }$$

By mean of Equations (1) and (2), the TIC area for each analyte after a single extraction on the sample can be used to estimate the total area. Consequently, a quantitation of the analytes can be carried out by the interpolation of the A_tot_ in a calibration curve obtained by a typical linear regression model. In this way, the method based on the multiple extractions allows the analyst to quantify compounds in samples with a simplified procedure, since a single extraction is sufficient for the calculation of factors and curves required to estimate analytes amounts [21].

The aim of the present work was to optimise and employ a proper MHS-SPME-GC-MS method, with the final goal to identify and quantify key volatile compounds responsible for white sturgeon (*A. transmontanus*) caviar flavour and to detect expected changes in their amounts during the ripening time.

## 2. Results and Discussion

### 2.1. MHS Extraction Method Development

**Figure 1:**
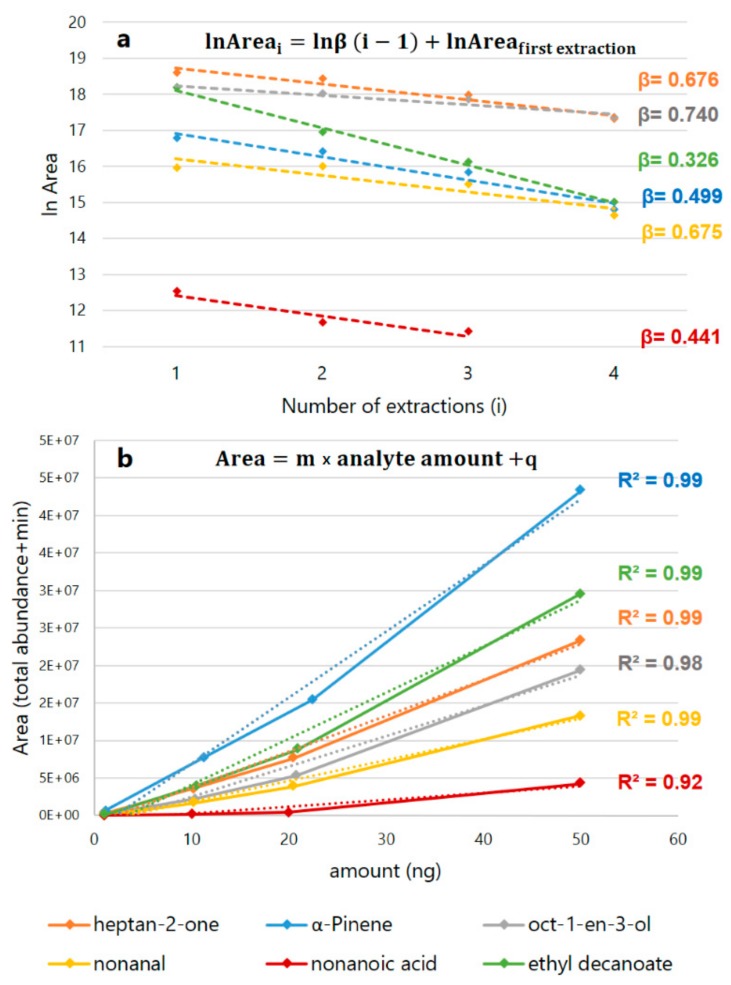
Linear regression plots employed in the determination of β values for each target compound, by means of the multiple extractions technique on calibration mixtures (**a**), and to the estimation of analytes’ total areas in samples, by mean of liquid injections of four different concentrations of calibration mixtures (**b**) (numerical data are shown in Table 1).

The curves, equations and calculation factors obtained by the development of the MHS-SPME techniques are shown in Table 1 and Figure 1.

**Figure 2:**
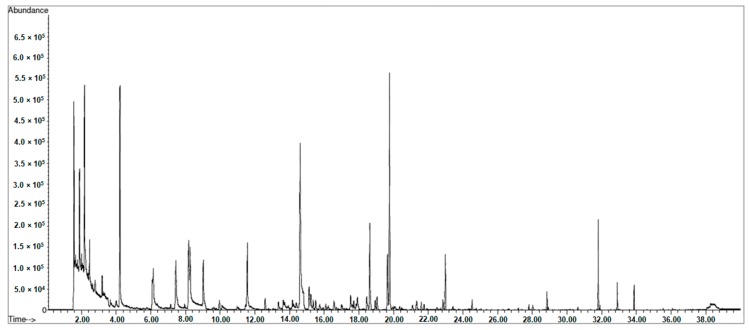
Illustrative TIC of volatile organic compounds in a caviar sample corresponding to a ripening time of 240 days (t4) by mean of MHS-SPME-GC-MS.

The development of the multiple extraction technique provided a good response when considering the exponential decay of target analytes during the consecutive extractions. With the only exception of ethyl decanoate (β = 0.326), the β factors obtained were included a range considered optimal for a correct estimation of the analytes’ total areas (0.4 < β < 0.95). A β value higher than 0.95, in fact, would indicate that the chromatographic area of the analyte under investigation appears unchanged even after several extractions, meaning that the amount of analyte the fibre can extract is meagre if compared to its total amount. Conversely, a β value lower than 0.4 would mean that the reduction of the chromatographic area among successive extractions is very consistent and that the analyte could be exhaustively extracted even by mean of a single extraction [22,23]. We obtained R^2^ ≥ 0.98 for all the curves obtained after the external calibration of the instrument by mean of liquid injection, with the exception of nonanoic acid that showed a R^2^ = 0.87. This phenomenon can been imputed to the low solubility of highly polar carboxylic acids in the non-polar stationary phase of the column (DB-5MS) employed in the chromatographic separation of compounds, resulting in peak fronting and in a low system sensitivity for nonanoic (as well as a higher LOD). In Figure 2, a TIC chromatogram of a representative sample analysed after 240 days of ripening (t4) is presented.

### 2.2. Caviar VOCs Identification and Quantification

Twenty-five key volatile compounds were detected with a good degree of certainty in eggs and caviar samples, showing a significant variability among different ripening times. The estimative quantitation of compounds by mean of the multiple extractions procedure provided reliable results. For many compounds, we found a great variability among samples collected at the same ripening time, reflecting considerable standard deviations within the same group (t0, t1, t2, t3, and t4). However, it has to be specified that such entity of data variability could be imputed to the fact that caviar analysed in this study was collected by the producer in cans of different dimensions (500 g or 1800 g) and adding slightly different concentrations of NaCl (3.6% or 3.8%), which could have influenced the ripening process and led to the huge variability. To overcome this problem, during the construction of the statistical model, we considered cans dimension and NaCl percentage as within-subject factors, in order to evaluate only the significance of the ripening time.

**Figure 3:**
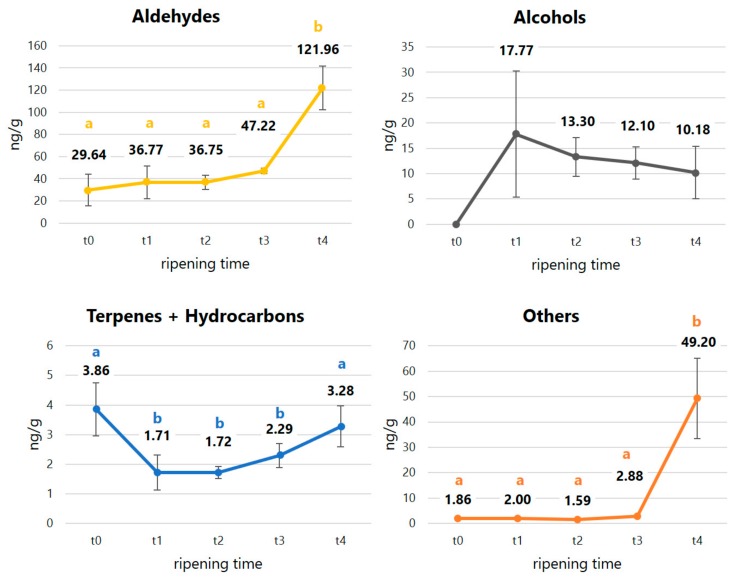
Development of the volatile compounds profile among t0 (raw eggs) and t4 (240 days) in caviar samples analysed by MHS-SPME-GC-MS.

VOCs detected in caviar samples were represented by aldehydes, alcohols, terpenes and non-terpenes hydrocarbons, one acid and one ketone, as shown in Table 2 and Figure 3.

Most of the volatile compounds found in fish products have been previously associated with the microbial and enzymatic activities occurring during the maturation of the products and with the lipoxygenases pathways acting against fatty acids [26].

The largest group of volatiles found in our work was represented by aldehydes. Several aldehydes have been previously found in different fresh and stored fish products [27,28,29,30,31,32], including eggs and caviar [9,33], showing mostly an important increase during the storage time [28,29,32]. Such aldehydes are considered aroma-active compounds in seafood since they contribute to the characteristic fish-like odour of fish products [12], also during the cold storage [34]. According to this, we found a significant increase in the total amount of aldehydes in caviar between t0 (29.64 ng/g) and t4 (121.96 ng/g). Aldehydes are primarily recognised as secondary unsaturated fatty acids (UFA) peroxidation products [31,35,36,37,38], formed by the action of several lipoxygenase systems on n3, n6 and n9 series UFA [31,39,40]. For instance, 15-lipoxygenase acts on n3 or n6 polyunsaturated fatty acids [31,39], mainly linoleic acid [40]; consequently, from the 13-hydroperoxide of linoleate, hexanal is produced. Octanal, nonanal and decanal are formed from autoxided n9 UFA, particularly oleic acid [28,35,41], while (*E*)-hex-2-enal and (2*E*,4*E*)-hepta-2,4-dienal originate from the oxidation of n3 PUFA [28], particularly eicosapentaenoic acid (EPA) and docosahexaenoic acid (DHA) [40]. The presence of such aldehydes in sturgeon caviar at higher concentration at t3 is certainly related to the breakdown of the radicals of the most representative fatty acids in the matrix during the ripening time. As evidenced by many authors, in fact, oleic acid, linoleic acid, EPA and DHA represent more than 50% of total fatty acids in caviar from farmed sturgeon [7,9,10,42]. In addition, we found 3-methylbutanal and 2-methylbutanal, which are generally considered as key spoilage indicators derived by microbial activity [43,44,45], a consequence of amino acid degradation [29]. The occurrence of many aldehydes, especially the branched and short chain ones, has been suggested to be associated with the breakdown of amino acids by several authors [46,47]. Particularly, 3-methylbutanal is thought to derive by the degradation of leucine [48], while 2-methylbutanal from isoleucine [41,46,49,50]. In the same way, 2-phenylacetaldehyde and 3-methylsulfanylpropanalderive are formed by the Strecker degradation breakdown of phenylalanine and methionine [51]. Generally, Strecker amino acids degradation is a process enhanced by high temperatures [52], thus the presence of such compounds in a fresh product such as raw caviar, stored at −2 °C, might suggest that other degradation pathways could have occurred leading to the formation of such compounds. This hypothesis is supported by the fact that several pathways, other than Maillard reaction, are involved in Strecker aldehydes formation. For instance, the presence of mild oxidising agents (such as metal catalysts) can lead to the oxidative decarboxylation of amino acids followed by hydrolysis of the imines also at ambient temperature [53]. Moreover, other authors previously detected Strecker aldehydes in fish tissues, even in cold storage conditions [50], [54,55,56], and, particularly in the case of 3-methylsulfanylpropanal, it showed a significant increase during the storage [28].

In the present work, we detected four alcohols, showing a variable trend within t0 and t4. Similar to aldehydes, alcohols in fish products are formed by the action of lipoxygenase on fatty acids (FA) [26] and by the decomposition of the secondary hydroperoxides of FA [57,58]. Particularly, it is known that pent-1-en-3-ol is formed by the action of 15-lypoxigenase on EPA and 12-lypoxygenase on arachidonic acid (ARA) while oct-1-en-3-ol derives by the enzymatic reaction of degradation of linoleic acid (LA) [28,37,40,55,59,60]. Oct-1-en-3-ol has been identified as one of the principal volatile alcohols in several seafoods [58,61,62] and previously found also in caviar [9]. Pent-1-en-3-ol, indeed, other than lipid peroxidation product, is known to be related to the microbial spoilage activity [31,63]. The absence of pent-1-en-3-ol in t0 and its presence in t1, t2, t3 and t4 samples is in good agreement with results reported by other authors [29,45,59,64]. In the same way, 3-methylbutan-1-ol and 2-ethylhexan-1-ol in fish products have been recognised as microbial spoilage compounds, deriving by the degradation of amino acids (mainly, valine) and lipids [45,48,63]. Other authors have previously detected these alcohols in raw tissues of many species of fish and seafood products [26,28,29,31,32,59,60,65,66,67,68], with a trend of increase during the storage [12,28,29,32,59,65]. For their characteristic marked production during the middle and later stages of fish products storage [68], volatile alcohols have been previously suggested as spoilage and oxidation indicators [39,59,69], also contributing to the off-odours in fish caused by the amino acids and lipid degradation [63].

Several odour-active terpene derivatives and two unsaturated hydrocarbons were identified in sturgeon eggs and caviar in this work. Several authors have previously found the same terpenes in fish products and suggested that this family of VOCs is most likely related to fish feed, deriving from algae or plants source [28,58,59,62,65,69,70]. Even in our case, we can hypothesise that such compounds reached sturgeon eggs via the food chain and that their presence did not suggested any significant influence of the ripening pathways occurring in caviar during the storage time. On the contrary, the unsaturated hydrocarbon pristane (IUPAC name: 2,6,10,14-tetramethylpentadecane), a common hydrocarbon originating from fossil and biogenic sources, is known to be present in aquatic environments and has been previously suggested to reach seafood products, included caviar, by means of the lipid autoxidation processes or from the decomposition of the carotenoids [9,61,71].

Finally, in our samples, we identified two compounds considered characteristic in fish [12,72]. Meagre amounts of nonanoic acid, ranging from 1.03 to 2.88 ng/g, were found in caviar during the entire storage period, even if without any statistical significance. On the contrary, an interesting, great arise of 3-hydroxy-2-butanone was detected just at the final stage (t4) of the storage time, reaching an amount of 48.17 ng/g. Nonanoic acid is considered one of the major compounds in the original seaweed by-product, deriving from the degradation of polyunsaturated fatty acids either by auto-oxidation or by the action of enzymes, representing a precursor to seafood flavours [12,73]. The presence of 3-hydrxoxy-2-butanone in fish products, indeed, has been related many times to the growth of microbial strains, e.g., by Ólafsdóttir et al. [65,72]. The significant increase of the amount of this compound in seafood products, even when cold storage, has led the authors to suggest this compound as an early indicator of spoilage, useful to monitor the loss of freshness.

The presence of several compounds derived from the lipid peroxidation processes leads us to suggest that, even if caviar were stored in controlled, strict conditions, the high amount unsaturated fatty acids could yield a relevant aptitude toward oxidation. The high unsaturation rate, in fact, could have balanced the reduction of lipid degradation due to the low storage temperature and operated by the antioxidant systems naturally active in the eggs.

## 3. Materials and Methods

### 3.1. Samples

Four White sturgeon (*Acipenser transmontanus*) egg samples and twelve caviar samples were provided by an Italian caviar company (Agroittica Lombarda SpA, Calvisano, BS, Italy). Each set of samples was collected at different stages of production: raw eggs (t0, *n* = 4), 60 days (t1, *n* = 4), 120 days (t2, *n* = 4), 180 days (t3, *n* = 2) and 240 days (t4, *n* = 4) of ripening, for a total of eighteen samples. The caviar analysed was salted with 3.6% or 3.8% of NaCl and stored in 500 or 1800 g cans at −2 °C, with the exception of t3 samples that only include caviar ripened in 500 g cans. Other caviar samples, used as matrix to optimise the analytical procedure, were purchased from the same company. For each sample, an aliquot of 5 g of raw matrix (eggs or caviar) was employed in the analysis without any treatment before VOCs extraction; each sample was analysed in triplicate.

### 3.2. SPME, GC and MS Parameters

The extraction of volatile compounds was performed by HS-SPME, using a multipurpose sampler MPS2 XL (Gerstel GmbH, Mulheim and der Ruhr, Germany) equipped with the SPME option, followed by GC-MS analysis. DVB/CAR/PDMS 1 cm SPME fibres were purchased by Supelco (Bellefonte, PA, USA) and used for the HS sampling. This fibre was chosen because of its capacity to extract a high number of VOCs, of different chemical species with different polarities and molecular weights. The bipolar compounds we expected to find in caviar samples, primarily aldehydes, ketones and alcohols, in fact, are known to be better extracted by fibres made of a combination of non-polar and polar materials [44]. Moreover, we expected to find VOCs in caviar samples at very low concentrations; the DVB/CAR/PDMS works by an adsorption mechanism that is strong and efficient, making this kind of device suitable for analysis on low concentrations compounds. The fibre was exposed to the calibration solutions or sample HS for 30 min at 60 °C. Extracted analytes were recovered by thermal desorption of the fibre into the injection port of the GC system at 250 °C for 1 min. The fibre was left in the injection port with the split valve open for 15 min for conditioning. The GC-MS system consisted of a 6890N Network GC system coupled to a 5973Network Mass Selective Detector (Agilent Technologies, Inc., Santa Clara, CA, USA). The column installed in the GC was a DB-5MS (30 m × 0.25 mm id, 0.25 μm film thickness) from Agilent Technologies. During the SPME desorbing phase, the injection port of the GC system was set in splitless mode; during the liquid injection of standard solutions, it was set in split mode (split ratio 1:100). A purge flow of 50 mL/min was set at 2 min to avoid an oversaturation of the MS ion source. The carrier gas was helium with a flow 1.0 mL/min and a pressure of 6.71 psi. The oven temperature program was as follows: from 35 °C (5 min) to 150 °C at 5 °C/min, and then from 150 °C to 260 °C at 10 °C/min (2 min). The mass detector operated in electron ionisation (EI) mode at 70 eV. The scan range of the MS was set to *m*/*z* 35-300 with a scanning rate of 5.19 scans/s. Data were acquired by Enhanced ChemStation (Agilent Technologies, Inc., Santa Clara, CA, USA).

### 3.3. Identification of the Volatiles

Key aroma compounds were experimentally selected by extractions performed on representative aliquots of eggs and caviar. Firstly, VOCs were tentatively identified by standard NIST MS library data, and then the identification of selected compound was performed by matching retention indices (RI) according to the theory by van den Dool and Kratz [24]. The LRI were calculated by retention times of a homologous series of *n*-alkane [25]. The series of *n*-alkanes C7 to C30 (1 mg/mL) for determination of RI was purchased by Supelco (Bellefonte, PA, USA). Mass spectra of authentic standards purchased from Sigma Aldrich (Milan, Italy), when available, were collected for VOCs identity confirmation (STD in Table 2). Standard mixtures adopted in identity confirmation were prepared in hexane as solvent at a 10 mg/mL concentration and stored refrigerated. Before the injection, solutions were diluted to a final concentration of 1 mg/mL in hexane and a volume of 1 μL was injected.

### 3.4. Quantification by Multiple-Extractions and External Calibration Approach

A standard mixture was made selecting one control compound for each family of target compounds detected by the extractions performed on the representative aliquots of eggs and caviar, according to the method of Bueno et al. [17]. The peaks that better arranged in the chromatogram to avoid coelutions were chosen: heptan-2-one for ketones, 1R-α-pinene for terpenes and unsaturated hydrocarbons, oct-1-en-3-ol for alcohols, nonanal for aldehydes, nonanoic acid for acids and ethyl decanoate for esters. All the analytical standards were purchased from Sigma Aldrich (Milan, Italy). The reference stock solution of target analytes was prepared in acetone as solvent at a 10 mg/mL concentration and stored in a vial under nitrogen at −18 °C for a maximum of four weeks. For the multiple-extraction method development, the reference stock solution was daily diluted and solutions were prepared fresh in 5 mL of HS-water (Sigma Aldrich, Milan, Italy), in order to cover a, for each analyte, the range of absolute amounts from 1 to 50 ng. Multiple extractions from the same calibration vial were performed setting the number of consecutive extractions at four, in order to achieve an almost-exhaustive extraction for all the analytes (a figure is provided in the Supplementary Materials). For the construction of the calibration equation, an external standard strategy was chosen to investigate the response of the instrumental equipment after known analyte amounts injections, as described by Serrano et al. [23]. Calibration solutions were prepared diluting the stock solution in hexane as solvent (Sigma Aldrich, Milan, Italy), covering four known concentration (100 μg/mL, 1 mg/mL, 2 mg/mL, and 5 mg/mL) in order to inject the corresponding total amount of 1, 10, 20, and 50 ng (injection volume 1 μL, split ratio 1:100). Each concentration was analysed in duplicate. The sensitivity of the detection system was measured by estimation of limit of detection (LOD) setting the signal to noise (S/N) ratio at 3 to the most diluted standard solution, according to other authors [19], [23].

### 3.5. Statistical Analysis

After data collection, the evaluation of the influence of storage time was performed by a univariate split-plot ANOVA for repeated measures. Significance was declared at *p* < 0.05 (*) and *p* < 0.01 (**). The statistical analysis was performed using JMP Pro 14 (SAS Institute Inc., Cary, NC, USA).

## 4. Conclusions

In this study, a method for the determination of caviar VOCs by mean of MHS-SPME coupled to GC–MS was developed and employed, showing the ability to identify and quantify VOCs in samples without any severe alteration of the matrix before the analysis and in a relatively short time. This method allowed a reliable estimation of the analytes’ quantities, solving the question of the non-exhaustive extraction due to the SPME working principle. The drawback in this kind of study remains the different analytes adsorption and partitioning behaviours that the authors think could have led to a competition among the components during the extraction phase and to interferences in the recovery rates. However, the results obtained predominantly show a trend in accordance to what is previously reported in the literature for the most of detected compounds. The relatively small number of compounds detected in caviar, if compared with the results obtained in previous studies on other fish products by headspace sampling techniques, may be because the storage conditions of analysed caviar samples were not so favourable for the microbial and enzymatic activities generally responsible for VOCs formation, as previously discussed. However, the results of this work mainly show the presence of several compounds that have been identified as characteristic of fish products, with some significant variations along different ripening time. The identification and the quantitative analysis of compounds responsible for caviar flavour described within this research represent an innovation in the field, adding knowledge and providing data almost missing in the literature to date. This may represent a substantial contribution to the available literature, beneficial to acquire a deep knowledge about this outstanding Italian product and also to protect and enhance its market.

## Figures and Tables

**Table 1:** Data obtained by the development of the multiple headspace-SPME (MHS-SPME) GC-MS method and by the external calibration curves. β factors were obtained by the logarithmic linear regression plot of the chromatographic areas recorded during the multiple extractions (shown in Figure 1a). The slope (m), the intercept (q) and the correlation coefficient (R^2^) are referred to the external calibration curves obtained by the injection of four known concentration of standards (shown in Figure 1b).

Target Family	Target Compound	β	Studied Range (ng)	m	q	R^2^	LOD(ng)
**Aldehydes**	nonanal	0.67463	1–50	286363	−1 × 10^6^	0.9834	0.71
**Alcohols**	oct-1-en-3-ol	0.74021	1–50	422066	−2 × 10^6^	0.9773	0.91
**Acids**	nonanoic acid	0.44139	1–50	84741	572724	0.8744	1.92
**Terpenes and hydrocarbons**	α-Pinene	0.49902	1–50	910076	−2 × 10^6^	0.9903	0.17
**Ketones**	heptan-2-one	0.67552	1–50	490916	−1 × 10^6^	0.991	0.46
**Esters**	ethyl decanoate	0.3256	1–50	636175	−2 × 10^6^	0.9843	1.02

**Table 2:** Volatile compounds profile of caviar analysed by MHS-SPME-GC-MS method.

	Volatile Compounds	Retention Time (min)	Mean of Identification ^1^	LRI	t0 Raw Eggs	t1 Caviar 60 Days	t2 Caviar 120 Days	t3 Caviar 180 Days	t4 Caviar 240 Days	Sign
					*n* = 4	*n* = 4	*n* = 4	*n* = 2	*n* = 4	
	**Aldehydes**									
**1**	3-methylbutanal	3.196	MS, STD, LRI	655	3.48 ± 1.96 ^A^	8.84 ± 5.88 ^A^	9.07 ± 3.74 ^A^	10.10 ± 1.73 ^A^	29.66 ± 7.33 ^B^	**
**2**	2-methylbutanal	3.345	MS, LRI	664	nd	2.58 ± 1.20 ^A^	3.20 ± 1.46 ^A^	3.48 ± 0.51 ^A^	11.09 ± 2.76 ^B^	**
**3**	hexanal	7.449	MS, STD, LRI	801	6.70 ± 3.63 ^A^	8.87 ± 2.86 ^A^	7.77 ± 6.49 ^A^	9.93 ± 1.46 ^A^	19.31 ± 12.64 ^B^	**
**4**	(E)-hex-2-enal	9.541	MS, STD, LRI	854	nd	nd	nd	0.59 ± 0.52 ^A^	1.36 ± 0.76 ^B^	**
**5**	3-methylsulfanylpropanal	11.611	MS, LRI	905	nd	1.49 ± 1.07 ^A^	1.72 ± 0.62 ^A^	2.29 ± 0.22 ^A^	7.66 ± 3.25 ^B^	**
**6**	benzaldehyde	13.642	MS, STD, LRI	960	2.06 ± 0.82 ^A^	1.60 ± 0.49 ^A^	1.27 ± 0.24 ^A^	2.19 ± 0.07 ^A^	4.57 ± 0.97 ^B^	**
**7**	octanal	15.226	MS, STD, LRI	1003	2.48 ± 1.07 ^A^	1.26 ± 0.31 ^BC^	1.03 ± 0.21 ^B^	1.11 ± 0.08 ^BC^	1.64 ± 0.22 ^C^	**
**8**	(2E,4E)-hepta-2,4-dienal	15.481	MS, STD, LRI	1010	nd	nd	nd	nd	0.75 ± 0.85	
**9**	2-phenylacetaldehyde	16.566	MS, LRI	1042	2.01 ± 0.97 ^A^	7.92 ± 5.21 ^A^	7.80 ± 3.33 ^A^	11.46 ± 0.67 ^A^	38.29 ± 14.96 ^B^	**
**10**	(E)-oct-2-enal	17.082	MS, LRI	1058	nd	nd	nd	nd	0.97 ± 1.06	
**11**	nonanal	18.630	MS, STD, LRI	1104	11.64 ± 7.00 ^A^	3.24 ± 1.22 ^BC^	3.88 ± 0.85 ^B^	4.85 ± 0.67 ^BC^	5.98 ± 1.22 ^B^	**
**12**	decanal	21.755	MS, STD, LRI	1205	1.28 ± 0.39 ^A^	0.96 ± 0.22 ^AB^	1.01 ± 0.12 ^AB^	1.21 ± 0.22 ^AB^	0.68 ± 0.54 ^B^	*
	∑aldehydes				29.64 ± 14.31 ^A^	36.77 ± 14.97 ^A^	36.75 ± 6.54 ^A^	47.22 ± 2.53 ^A^	121.96 ± 19.80 ^B^	**
	**Alcohols**									
**13**	pent-1-en-3-ol	3.675	MS, LRI	682	nd	2.00 ± 0.52	1.96 ± 0.51	2.23 ± 0.29	2.91 ± 2.56	
**14**	3-methylbutan-1-ol	5.126	MS, STD, LRI	734	nd	nd	nd	nd	4.95 ± 6.32	
**15**	oct-1-en-3-ol	14.423	MS, STD, LRI	981	nd	2.07 ± 0.68	1.52 ± 0.70	1.46 ± 0.07	1.35 ± 1.50	
**16**	2-ethylhexan-1-ol	16.088	MS, LRI	1028	nd	13.70 ± 11.93	9.82 ± 3.84	8.41 ± 2.92	nd	
	∑alcohols				nd	17.77 ± 12.45	13.30 ± 3.82	12.10 ± 3.21	9.22 ± 5.56	
	**Terpenes and hydrocarbons**									
**17**	α-pinene	12.606	MS, STD, LRI	932	0.95 ± 0.44	0.97 ± 0.50	0.73 ± 0.14	0.66 ± 0.04	1.11 ± 0.40	
**18**	3-carene	15.374	MS, STD, LRI	1007	nd	nd	0.44 ± 0.18	0.57 ± 0.03	0.36 ± 0.28	
**19**	1,2,3-trimethylbenzene	15.760	MS, STD, LRI	1018	0.66 ± 0.11 ^A^	0.13 ± 0.23 ^B^	nd	0.35 ± 0.31 ^a^	nd	**
**20**	limonene	16.103	MS, STD, LRI	1029	0.63 ± 0.05	nd	nd	nd	0.60 ± 0.03	
**21**	β-ocimene	16.700	MS, STD, LRI	1047	0.44 ± 0.30	nd	nd	nd	nd	
**22**	caryophyllene	27.741	MS, STD, LRI	1423	0.14 ± 0.27	nd	nd	nd	nd	
**23**	pristane	32.902	MS, LRI	1704	1.04 ± 0.43 ^AC^	0.62 ± 0.06 ^B^	0.56 ± 0.05 ^B^	0.71 ± 0.03 ^AB^	1.20 ± 0.22 ^C^	**
	∑terpenes and hydrocarbons				3.86 ± 0.90 ^A^	1.71 ± 0.59 ^B^	1.72 ± 0.20 ^B^	2.29 ± 0.41 ^B^	3.28 ± 0.69 ^A^	**
	**Other compounds**									
**24**	2-butanone, 3-hydroxy	4.797	MS, LRI	724	nd	nd	nd	nd	48.17 ± 16.87	
**25**	nonanoic acid	23.424	MS, STD, LRI	1263	1.86 ± 0.33	2.00 ± 0.60	1.59 ± 0.13	2.88 ± 0.12	1.03 ± 1.65	
	∑other compounds				1.86 ± 0.33 ^A^	2.00 ± 0.60 ^A^	1.59 ± 0.13 ^A^	2.88 ± 0.12 ^A^	49.20 ± 15.81 ^B^	**

**^1^** Comparison with MS spectra obtained by NIST library (MS), comparison with retention time and spectra of authentic reference compounds (STD), comparison with Linear Retention Indices (LRI) by van den Dool and Kratz [24] for a DB-5MS capillary column, calculated by a *n*-alkanes series [25] found in the literature. ^A,B,C^= values within the same row associated with different letters are significantly different (* *p* < 0.05; ** *p* < 0.01). Quantitative data are expressed as ng/g of sample (mean ± standard deviation)

## Supplementary Materials

The following are available online at https://www.mdpi.com/1420-3049/25/5/1074/s1; Figure S1. Illustrative multiple headspace solid phase microextraction protocol on a calibration mixture using an automated MPS multipurpose sampler (Gerstel Mullheim a/d Ruhr, Germany).

## References

[1] Codex Alimentarius Codex Standard (2013). CODEX STAN 291–2010 Page 2 of 4. http://www.fao.org/fao-who-codexalimentarius/codex-texts/list-standards/en/.

[2] Bronzi P., Chebanov M., Michaels J.T., Wei Q., Rosenthal H., Gessner J. (2019). Sturgeon meat and caviar production: Global update 2017. J. Appl. Ichthyol..

[3] CITES Convention on International Trade in Endangered Species of Wild Fauna and Flora. https://www.cites.org/eng/disc/text.php.

[4] Fain S.R., Straughan D.J., Hamlin B.C., Hoesch R.M., LeMay J.P. (2013). Forensic genetic identification of sturgeon caviars traveling in world trade. Conserv Genet..

[5] Rehbein H., Molkentin J., Schubring R., Lieckfeldt D., Ludwig A. (2008). Development of advanced analytical tools to determine the origin of caviar. J. Appl Ichthyol..

[6] Pappalardo A.M., Petraccioli A., Capriglione T., Ferrito V. (2019). From Fish Eggs to Fish Name: Caviar Species Discrimination by COIBar-RFLP, an Efficient Molecular Approach to Detect Fraud in the Caviar Trade. Molecules.

[7] Gessner J., Würtz S., Kirschbaum F., Wirth M. (2008). Biochemical composition of caviar as a tool to discriminate between aquaculture and wild origin. J. Appl Ichthyol..

[8] Wirth M., Kirschbaum F., Gessner J., Williot P., Patriche N., Billard R. (2002). Fatty acid composition in sturgeon caviar from different species: Comparing wild and farmed origins. Int Rev. Hydrobiol..

[9] Caprino F., Moretti V.M., Bellagamba F., Turchini G.M., Busetto M.L., Giani I., Paleari M.A., Pazzaglia M. (2008). Fatty acid composition and volatile compounds of caviar from farmed white sturgeon (Acipenser transmontanus). Anal. Chim Acta..

[10] Wirth M., Kirschbaum F., Gessner J., Krüger A., Patriche N., Billard R. (2000). Chemical and biochemical composition of caviar from different sturgeon species and origins. Nahrung - Food..

[11] (1991). Guidelines for the Sensory Evaluation of Fish and Shellfish in Laboratories.

[12] Josephson D.B., Lindsay R.C. (1986). Enzymic Generation of Volatile Aroma Compounds from Fresh Fish. Biogeneration of Aromas.

[13] Arthur C.L., Pawliszyn J. (1990). Solid Phase Microextraction with Thermal Desorption Using Fused Silica Optical Fibers. Anal. Chem..

[14] Zhang Z., Pawliszyn J. (1993). Headspace Solid-Phase Microextraction. Anal. Chem..

[15] Ouyang G., Pawliszyn J. (2008). A critical review in calibration methods for solid-phase microextraction. Anal. Chim Acta..

[16] García-Vico L., Belaj A., Sánchez-Ortiz A., Martínez-Rivas J.M., Pérez A.G., Sanz C. (2017). Volatile Compound Profiling by HS-SPME/GC-MS-FID of a Core Olive Cultivar Collection as a Tool for Aroma Improvement of Virgin Olive Oil. Molecules.

[17] Bueno M., Resconi V.C., Campo M.M., Ferreira V., Escudero A. (2019). Development of a robust HS-SPME-GC-MS method for the analysis of solid food samples. Analysis of volatile compounds in fresh raw beef of differing lipid oxidation degrees. Food Chem..

[18] Cordero C., Guglielmetti A., Sgorbini B., Bicchi C., Allegrucci E., Gobino G., Baroux L., Merle P. (2019). Odorants quantitation in high-quality cocoa by multiple headspace solid phase micro-extraction: Adoption of FID-predicted response factors to extend method capabilities and information potential. Anal. Chim Acta..

[19] Costa R., Tedone L., De Grazia S., Dugo P., Mondello L. (2013). Multiple headspace-solid-phase microextraction: An application to quantification of mushroom volatiles. Anal. Chim Acta..

[20] Kolb B. (1982). Multiple headspace extraction-A procedure for eliminating the influence of the sample matrix in quantitative headspace, gas chromatography. Chromatographia.

[21] Kolb B., Ettre L.S. (2006). Static Headspace-Gas. Chromatography. Theory and Practice.

[22] Tena M.T., Carrillo J.D. (2007). Multiple solid-phase microextraction: Theory and applications. TrAC - Trends Anal. Chem..

[23] Serrano E., Beltrán J., Hernández F. (2009). Application of multiple headspace-solid-phase microextraction followed by gas chromatography-mass spectrometry to quantitative analysis of tomato aroma components. J. Chromatogr A..

[24] Van Den Dool H., Kratz P.D. (1963). A Generalization of the Retention Index System Including Linear Temperature Programmed Gas-Liquid Partition Chromatography. J. Chromatogr A..

[25] Kovàts E. (1958). Characterization of organic compounds by gas chromatography. Part 1. Retention indices of aliphatic halides, alcohols, aldehydes and ketones..

[26] Duflos G., Coin V.M., Cornu M., Antinelli J.F., Malle P. (2006). Determination of volatile compounds to characterize fish spoilage using headspace/mass spectrometry and solid-phase microextraction/gas chromatography/mass spectrometry. J. Sci Food Agric..

[27] Iglesias J., Gallardo J.M., Medina I. (2010). Determination of carbonyl compounds in fish species samples with solid-phase microextraction with on-fibre derivatization. Food Chem..

[28] Prost C., Hallier A., Cardinal M., Serot T., Courcoux P. (2004). Effect of Storage Time on Raw Sardine (Sardina pilchardus) Flavor and Aroma Quality. J. Food Sci..

[29] Aro T., Tahvonen R., Koskinen L., Kallio H. (2003). Volatile compounds of Baltic herring analysed by dynamic headspace sampling-gas chromatography-mass spectrometry. Eur Food Res. Technol..

[30] Milo C., Grosch W. (1993). Changes in the Odorants of Boiled Trout (Salmo fario) As Affected by the Storage of the Raw Material. J. Agric. Food Chem..

[31] Josephson D.B., Lindsay R.C., Stuiber D.A. (1984). Biogenesis of lipid-derived volatile aroma compounds in the emerald shiner (Notropis atherinoides). J. Agric. Food Chem..

[32] Iglesias J., Medina I., Bianchi F., Careri M., Mangia A., Musci M. (2009). Study of the volatile compounds useful for the characterisation of fresh and frozen-thawed cultured gilthead sea bream fish by solid-phase microextraction gas chromatography-mass spectrometry. Food Chem..

[33] Moretti V.M., Vasconi M., Caprino F., Bellagamba F. (2017). Fatty Acid Profiles and Volatile Compounds Formation During Processing and Ripening of a Traditional Salted Dry Fish Product. J. Food Process. Preserv..

[34] Ólafsdóttir G. (2005). Volatile Compounds As Quality Indicators In Chilled Fish: Evaluation Of Microbial Metabolites By An Electronic Nose. Ph.D. Thesis.

[35] Frankel E.N. (2005). Foods. Lipid Oxidation.

[36] Grosh W., Morton I.D., MacLeod A.J. (1982). Lipid degradation products and flavours. Food Flavours. Part A- Introduction.

[37] Josephson D.B., Lindsay R.C., Stuiber D.A. (1983). Identification of Compounds Characterizing the Aroma of Fresh Whitefish (*Coregonus clupeaformis*). J. Agric. Food Chem..

[38] Karahadian C., Lindsay R.C. (1989). Evaluation of compounds contributing characterizing fishy flavors in fish oils. J. Am. Oil Chem Soc..

[39] Lindsay R.C. (1990). Fish flavors. Food Rev. Int..

[40] Kawai T., Sakaguchi M. (1996). Fish flavor. Crit Rev. Food Sci Nutr..

[41] Jónsdóttir R., Ólafsdóttir G., Chanie E., Haugen J.E. (2008). Volatile compounds suitable for rapid detection as quality indicators of cold smoked salmon (*Salmo salar*). Food Chem..

[42] Lopez A., Vasconi M., Bellagamba F., Mentasti T., Moretti V.M.M. (2020). Sturgeon meat and caviar quality from different cultured species. Fishes.

[43] Joffraud J.J., Leroi F., Roy C., Berdagué J.L. (2001). Characterisation of volatile compounds produced by bacteria isolated from the spoilage flora of cold-smoked salmon. Int J. Food Microbiol..

[44] Balasubramanian S., Panigrahi S. (2011). Solid-Phase Microextraction (SPME) Techniques for Quality Characterization of Food Products: A Review. Food Bioprocess. Technol..

[45] Jørgensen L.V., Huss H.H., Dalgaard P. (2001). Significance of volatile compounds produced by spoilage bacteria in vacuum-packed cold-smoked salmon (*Salmo salar*) analyzed by GC-MS and multivariate regression. J. Agric. Food Chem..

[46] Ardö Y. (2006). Flavour formation by amino acid catabolism. Biotechnol Adv..

[47] Giri A., Osako K., Okamoto A., Ohshima T. (2010). Olfactometric characterization of aroma active compounds in fermented fish paste in comparison with fish sauce, fermented soy paste and sauce products. Food Res. Int..

[48] Ólafsdóttir G., Jónsdóttir R., Nollet L.L.M., Toldrà F. Volatile Aroma Compounds in Fish. Handbook fo Seafood and Seafood Products Analysis.

[49] Pripi-Nicolau L., De Revel G., Bertrand A., Maujean A. (2000). Formation of flavor components by the reaction of amino acid and carbonyl compounds in mild conditions. J. Agric. Food Chem..

[50] Milo C., Grosch W. (1995). Detection of Odor Defects in Boiled Cod and Trout by Gas Chromatography–Olfactometry of Headspace Samples. J. Agric. Food Chem..

[51] Weenen H., Van Der Ven J.G.M. (2001). The formation of strecker aldehydes. ACS Symp Ser..

[52] Belitz H.D., Grosch W., Schieberle P. (2009). Food Chemistry.

[53] Yaylayan V.A. (2003). Recent Advances in the Chemistry of Strecker Degradation and Amadori Rearrangement: Implications to Aroma and Color Formation. Nippon Shokuhin Kagaku Kogaku Kaishi.

[54] Aro T., Brede C., Manninen P., Kallio H. (2002). Determination of semivolatile compounds in Baltic herring (*Clupea harengus membras*) by supercritical fluid extraction-supercritical fluid chromatography-gas chromatography-mass spectrometry. J. Agric. Food Chem..

[55] Selli S., Cayhan G.G. (2009). Analysis of volatile compounds of wild gilthead sea bream (*Sparus aurata*) by simultaneous distillation-extraction (SDE) and GC-MS. Microchem J..

[56] Selli S., Rannou C., Prost C., Robin J., Serot T. (2006). Characterization of aroma-active compounds in rainbow trout (I) eliciting an off-odor. J. Agric. Food Chem..

[57] Tanchotikul U., Hsieh T.C.Y. (1989). Volatile Flavor Components in Crayfish Waste. J. Food Sci..

[58] Tanchotikul U., Hsieh T.C.Y. (1991). Analysis of Volatile Flavor Components in Steamed Rangia Clam by Dynamic Headspace Sampling and Simultaneous Distillation and Extraction. J. Food Sci..

[59] Alasalvar C., Taylor K.D.A., Shahidi F. (2005). Comparison of volatiles of cultured and wild sea bream (*Sparus aurata*) during storage in ice by dynamic headspace analysis/gas chromatography-mass spectrometry. J. Agric. Food Chem.

[60] Hsieh R.J., Kinsella J.E. (1989). Lipoxygenase Generation of Specific Volatile Flavor Carbonyl Compounds in Fish Tissues. J. Agric. Food Chem..

[61] Spurvey S., Pan B.S., Shahidi F., Shahidi F. (1998). Flavour of shellfish. Flavor of Meat, Meat Products and Seafoods.

[62] Vejapham W., Hsieh T.C.Y., Williams S.S. (1988). Volatile Flavor Components from Boiled Crayfish (*Procambarus clarkii*) Tail Meat. J. Food Sci..

[63] Miller A., Scanlan R.A., Lee J.S., Libbey L.M., Morgan M.E. (1973). Volatile compounds produced in sterile fish muscle (Sebastes melanops) by Pseudomonas perolens. Appl Microbiol..

[64] Nordvi B., Langsrud O., Egelandsdal B., Slinde E., Vogt G., Gutierrez M., Olsen E. (2007). Characterization of volatile compounds in a fermented and dried fish product during cold storage. J. Food Sci..

[65] Olafsdottir G., Jonsdottir R., Lauzon H.L., Luten J., Kristbergsson K. (2005). Characterization of volatile compounds in chilled cod (*Gadus morhua*) fillets by gas chromatography and detection of quality indicators by an electronic nose. J. Agric. Food Chem..

[66] Laursen B.G., Leisner J.J., Dalgaard P. (2006). Carnobacterium Species: Effect of metabolic activity and interaction with Brochothrix thermosphacta on sensory characteristics of modified atmosphere packed shrimp. J. Agric. Food Chem..

[67] Fall P.A., Leroi F., Cardinal M., Chevalier F., Pilet M.F. (2010). Inhibition of Brochothrix thermosphacta and sensory improvement of tropical peeled cooked shrimp by Lactococcus piscium CNCM I-4031. Lett. Appl. Microbiol..

[68] Edirisinghe R.K.B., Graffham A.J., Taylor S.J. (2007). Characterisation of the volatiles of yellowfin tuna (*Thunnus albacares*) during storage by solid phase microextraction and GC-MS and their relationship to fish quality parameters. Int J. Food Sci Technol..

[69] Iglesias J., Medina I. (2008). Solid-phase microextraction method for the determination of volatile compounds associated to oxidation of fish muscle. J. Chromatogr A..

[70] Zhang Z., Li G., Luo L., Chen G. (2010). Study on seafood volatile profile characteristics during storage and its potential use for freshness evaluation by headspace solid phase microextraction coupled with gas chromatography-mass spectrometry. Anal. Chim Acta..

[71] Turchini G.M., Giani I., Caprino F., Moretti V.M., Valfrè F. (2004). Discrimination of origin of farmed trout by means of biometrical parameters, fillet composition and flavor volatile compounds. Ital. J. Anim Sci..

[72] Olafsdottir G., Nesvadba P., Di Natale C., Careche M., Oehlenschläger J., Tryggvadóttir S.V., Schubring R., Kroeger M., Heia K., Esaiassen M. (2003). Multisensor for fish quality determination. Trends Food Sci Technol..

[73] Le Pape M.-A., Grua-Priol J., Prost C., Demaimay M. (2007). Optimization of Dynamic Headspace Extraction of the Edible Red Algae Palmaria palmata and Identification of the Volatile Components. J. Agric. Food Chem.

