# Supplementary material for: Volatile Organic Compounds Profile in White Sturgeon (Acipenser transmontanus) Caviar at Different Stages of Ripening by Multiple Headspace Solid Phase Microextraction
Source: Molecules. 2020 Feb 27;25(5):1074. doi: 10.3390/molecules25051074 (PMC7179139; doi:10.3390/molecules25051074)

Figure S1. Illustrative Multiple Headspace Solid Phase Micro Extraction protocol on a calibration mixture using an automated MPS multipurpose sampler (Gerstel Mullheim a/d Ruhr, Germany)

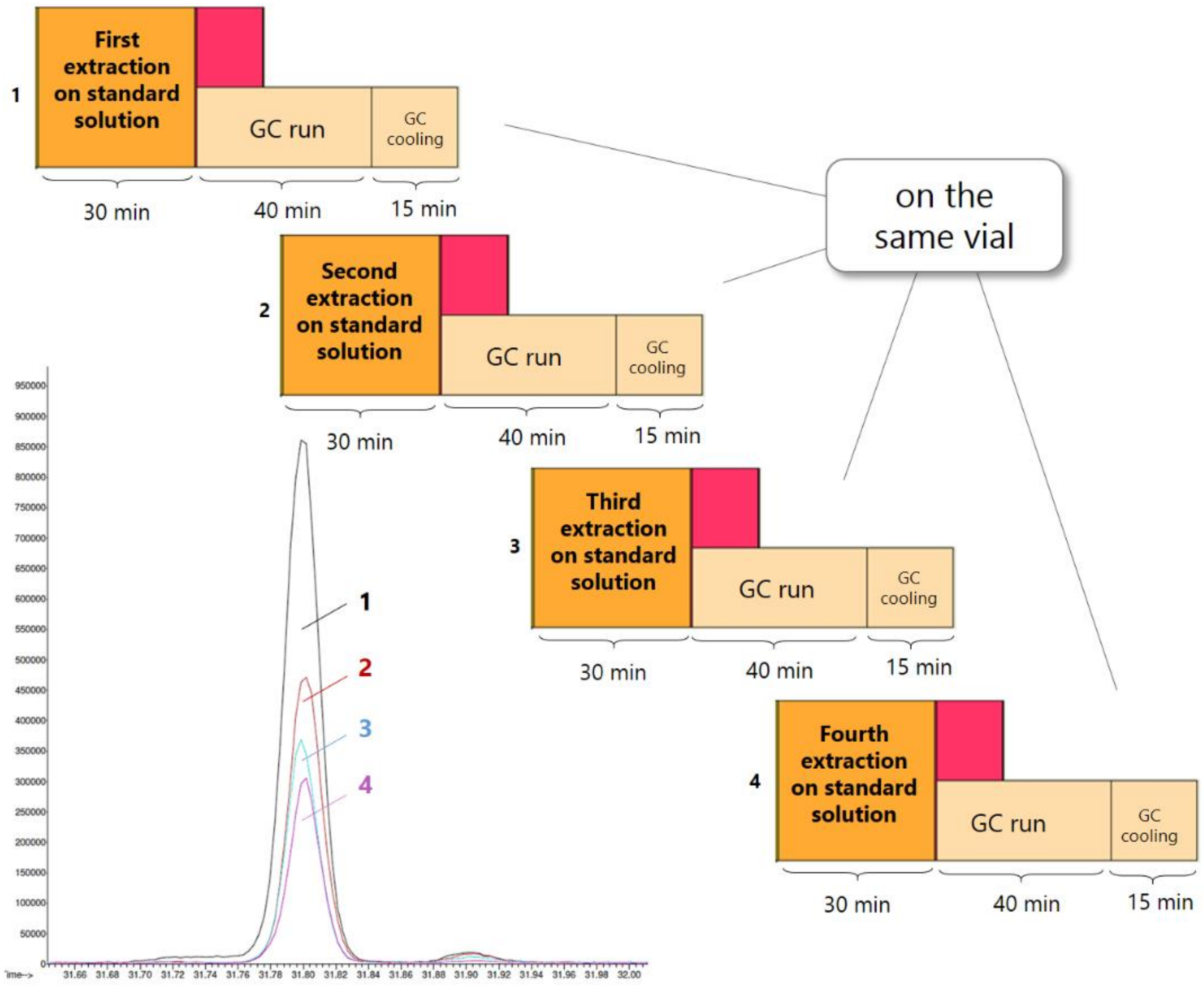

Supplement: Supplementary file 1 [file molecules-25-01074-s001.pdf]
